# Quercetin modulates the gut microbiota as well as the metabolome in a rat model of osteoarthritis

**DOI:** 10.1080/21655979.2021.1969194

**Published:** 2021-09-05

**Authors:** Haifeng Lan, Wei Hong, Dongyang Qian, Fang Peng, Haiqing Li, Chunxiao Liang, Min Du, Jinlan Gu, Junxuan Mai, Bo Bai, Gongyong Peng

**Affiliations:** a Department of Orthopaedic Surgery, The Third Affiliated Hospital of Guangzhou Medical University, Guangzhou, Guangdong, China; b The Division of Pulmonary and Critical Care Medicine, Guangzhou Institute of Respiratory Health, National Clinical Research Center for Respiratory Disease, State Key Laboratory of Respiratory Disease, National Center for Respiratory Medicine, the First Affiliated Hospital of Guangzhou Medical University, Guangzhou, Guangdong, China; c Gmu-gibh Joint School of Life Sciences, Guangzhou Medical University, Guangzhou, Guangdong, China; d Department of Orthopaedics, The First Affiliated Hospital, Guangzhou Medical University/Guangdong Key Laboratory of Orthopaedic Technology and Implant Materials, Guangzhou, Guangdong, China; e Department of Critical Care Medicine, The Third Affiliated Hospital of Guangzhou Medical University Guangzhou, Guangdong, China; f Department of Thoracic Medicine, Shenzhen Second People’s Hospital, the First Affiliated Hospital of Shenzhen University, Shenzhen, Guangdong, China

**Keywords:** Osteoarthritis, quercetin, gut microbiota, metabolome, regulation

## Abstract

Although the mechanism of osteoarthritis (OA) has been widely studied and the use of quercetin for OA therapy is well documented, the relevant characteristics of the microbiome and metabolism remain unclear. This study reports changes in the gut microbiota and metabolism during quercetin therapy for OA in a rat model and provides an integrative analysis of the biomechanism. In this study, the rats were categorized into 3 different groups: the OA model, quercetin treatment, and control groups. The OA rats was conducted using a monoiodoacetate (MIA) injection protocol. The rats in the quercetin group received daily intragastric administration of quercetin from day 1 to day 28. Stool samples were collected, and DNA was extracted. We used an integrated approach that combined the sequencing of whole 16S rRNA, short-chain fatty acid (SCFA) measurements and metabolomics analysis by mass spectrometry (MS) to characterize the functional impact of quercetin on the gut microbiota and metabolism in a rat model of OA. The use of quercetin partially abrogated intestinal flora disorder and reversed fecal metabolite abnormalities. Compared with the control rats, the OA rats showed differences at both the class level (*Clostridia, Bacteroidia*, and *Bacilli*) and the genus level (*Lactobacillus* and *unidentified Ruminococcaceae*). Acetic acid, propionic acid and 24 metabolites were significantly altered among the three groups. However, the changes were significantly abrogated in quercetin-treated OA rats. Consequently, this study provided important evidence regarding perturbations of the gut microbiome and the function of these changes in a potential new mechanism of quercetin treatment.

## Graphical ABSTRACT

**Figure:**
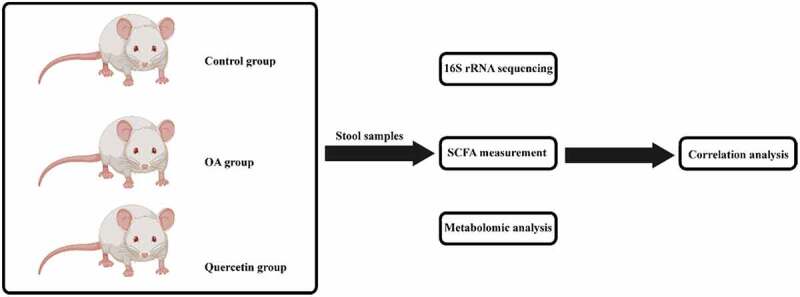

## Introduction

Osteoarthritis (OA) is one of the leading causes of disability and affects millions of people of all ages worldwide [1]. Due to the involvement of genetic, biological, and biomechanical components, OA is complicated and multifactorial, with etiological factors commonly found to be joint specific [2]. Despite the frequent use of joint replacement in symptomatic end-stage disease and its effectiveness, poor function and limited lifespans of prostheses are major concerns. Despite the option of joint replacement surgery, OA is commonly considered an incurable disease. However, quercetin, an important flavonoid, has been reported to affect the prevention and treatment of OA [3,4].

Quercetin exhibits antioxidative, anti-inflammatory, and antiosteoporotic effects. Some studies also found that quercetin significantly impacts the intestinal environment and subsequently affects the regulation of the gut microbiota. However, quercetin has also been considered a prebiotic [5]. In our opinion, one important factor that needs to be taken into consideration is the antimicrobial effect of quercetin, which may potentially be the critical factor that influences the microorganisms themselves. In human beings, microorganisms from both food pathogenic species (such as *Staphylococcus aureus* [6–9], *Escherichia coli* [10,11], *Salmonella* [12] *Listeria monocytogenes* [13], and *Vibrio parahaemolyticus* [14]) and clinically important hospital- or community-associated pathogens (‘ESKAPE’ including MRSA [15–17], MRCNS [18,19], Enterococcus [20], *Pseudomonas aeruginosa* [21,22] and *K. pneumoniae* [23,24]) may colonize the human microbiota. Both the prebiotic and antimicrobial effects of quercetin may eventually influence such microbiota, especially gut microbiota, which is closely linked with human disease. The intestine is perpetually exposed to gut microbes and their metabolites, which leads to a close correlation between the intestine and human health. Most studies on this relationship have compared the microbial species from the gut microbiota between a diseased group and a healthy group. A correlation between gut microbiota dysbiosis and OA has previously been reported [25,26]. A considerable amount of research on OA links OA to the gut microbiome, but further studies on the gut microbiome in OA are required.

Accordingly, this study aimed to elucidate possible mechanisms based on the correlation between the intestinal microbiome and the fecal metabolome using multiple omics analysis in OA rats. The impact of quercetin on the production of short-chain fatty acids (SCFAs) was also evaluated. These results provide objective evidence that quercetin functions as a drug for the treatment of OA.

## Materials and methods

### Rats and treatments

Quercetin and monoiodoacetate (MIA) were purchased commercially (Sigma-Aldrich). The experimental procedure was started 7 days after adaptive feeding. All animal procedures were approved by the Committee for the Institutional Care and Use of Animals of Curegenix Corporation, Ltd. (YSDW201911056).

The rats were categorized into 3 different groups, including the OA model, quercetin treatment (100 mg/kg, i.g., q.d.), and control groups. MIA-induced OA modeling in the rats was performed as previously described [27]. The animals were anesthetized using isoflurane (2.5%), and the depth of anesthesia was checked by toe pinch followed by a single intra-articular injection of 1 mg of MIA or an equivalent volume of phosphate-buffered saline (PBS) as a control. Rats in the quercetin-treated groups received intragastric administration once daily from day 1 to day 28. All rats were sacrificed 29 days postsurgery. The rats were weighed twice weekly.

### 16S rRNA gene sequencing and analysis

Fresh stool samples from the rats were collected and immediately stored at −80°C for subsequent DNA extraction. High-throughput sequencing and analysis of the fecal microbial 16S rRNA were performed according to previous studies **[**28**]**. Extracted DNA was sequenced using the Illumina HiSeq 2500 platform (Illumina, CA, USA), and the V3-V4 hypervariable region of the 16S rRNA gene was targeted with the primers 341 F (5ʹ-CCTAYGGGRBGCASCAG-3ʹ) and 806 R (5ʹ- GGACTACNNGGGTATCTAAT-3ʹ). Sequence assembly, quality control, and clustering were performed using Uparse v7.0.1001 software (http://www.drive5.com/uparse/).

### SCFA measurements

Quantification of SCFAs from the stool samples of rats was performed as described previously **[**29**]**. Fecal SCFAs were quantified using an Agilent 7890A gas chromatograph coupled with an Agilent 5975 C mass spectrometric detector (Agilent Technologies, USA) equipped with an HP-5 MS column (0.25 × 30 mm, 0.25-μm particle size) (Suzhou Bionovogene Co., Ltd) as described previously. Helium was used as a carrier gas at a constant flow rate of 1 mL/min. The initial oven temperature was held at 60°C for 5 min, increased to 250°C at a rate of 10°C/min, and finally held at this temperature for 5 min. The temperatures of the front inlet, transfer line and electron impact (EI) ion source were set as 280, 250 and 230°C, respectively. Data handling was performed using an Agilent MSD ChemStation (E.02.00.493, Agilent Technologies, Inc., USA).

### Metabolomic analysis

The samples were analyzed using a Thermo Ultimate 3000 system (Suzhou Bionovogene Co., Ltd, Suzhou, China) according to the methods of previous studies **[**30**]**. The instrument was equipped with an ACQUITY UPLC® HSS T3 (150 × 2.1 mm, 1.8 μm, Waters, Milford, CT, USA), and the column was maintained at 40°C. The autosampler temperature and flow rate were 4°C and 0.25 mL/min, respectively. Gradient elution of the analytes was performed with 0.1% formic acid in water and 0.1% formic acid in acetonitrile.

The ESI-MSn experiments were performed in a Thermo Q-Exactive mass spectrometer (Bremen, Germany). The spray voltages were 3.8 and 2.5 kV in positive and negative ion modes, respectively. The capillary temperature was 325°C. The full scan was performed with a resolution of 60,000, and the mass range was 89–1000 m/z. Data-dependent acquisition tandem mass spectrometry (MS/MS) experiments were performed using a collision-induced degradation (CID) scan with a collision voltage of 30 eV. Dynamic exclusion was used to remove unnecessary MS/MS data, and the exclusion duration was set to 15 s.

### Correlation analysis of features of the gut microbiota and host metabolome

Pearson correlation analysis was used to reveal the correlation between the gut microbiome and host metabolome using the Cytoscape software coNet plug-in, and the correlation coefficient and P-value threshold were not set. P < 0.05 was regarded as statistically significant, P < 0.01 was regarded as very significant, and P < 0.001 was regarded as extremely significant. A heatmap was used to show the correlation between the gut microbiome and the host metabolome.

### Statistical analysis

Data are presented as the means ± standard deviations. Differences between groups were analyzed by one-way analysis of variance (ANOVA). Statistical significance was determined at P < 0.05. All statistical analyses were performed using SPSS 16.0 (SPSS, Chicago, IL, United States). The data are graphically presented using GraphPad Prism 7.

## Results

### Quercetin modulated changes in gut microbiota features in OA rats

**Figure 1.**
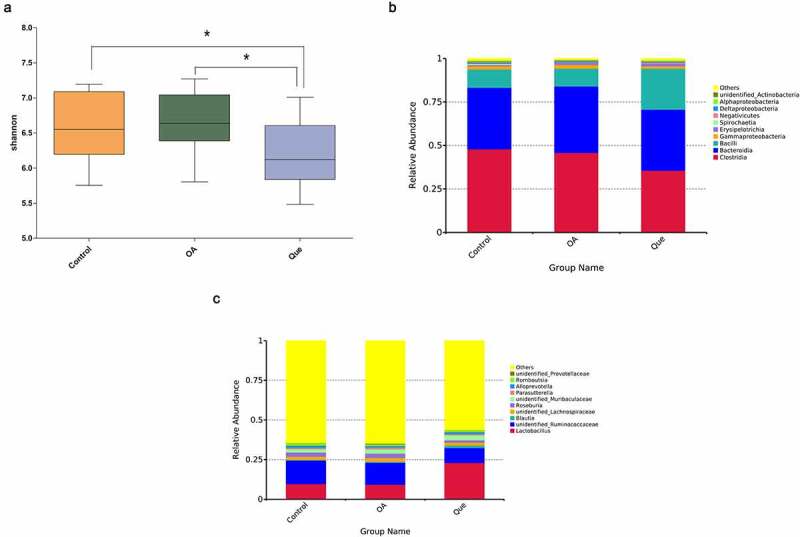
Intestinal microbial changes after quercetin treatment in OA rats. (a) Alpha diversity was evaluated using the Shannon index. Average relative abundances of dominant bacterial classes (b) and genera (c) in the intestine under quercetin treatment

An increasing number of studies have addressed the close link between changes in the gut microbiome composition and quercetin **[**31–33**]**. To elucidate this association with quercetin, gut microbiota analysis was performed by 16S rRNA sequencing. Alpha diversities revealed significant discrepancies between the gut microbiota species compositions in the quercetin group and the other groups (Figure 1a). Diversity in the gut microbiota decreased after treatment with quercetin, which indicated a meaningful modulation of the intestinal flora by quercetin. Compared with the control rats, OA rats showed differences at both the class and genus levels. At the class level, altered intestinal flora (*Clostridia, Bacteroidia*, and *Bacilli*) were observed in the three groups (Figure 1b). At the genus level, increased *Lactobacillus* and decreased *unidentified Ruminococcaceae* were observed following quercetin treatment (Figure 1c).

### Quercetin increased SCFA levels in the fecal intestinal metabolites of OA rats

**Figure 2.**
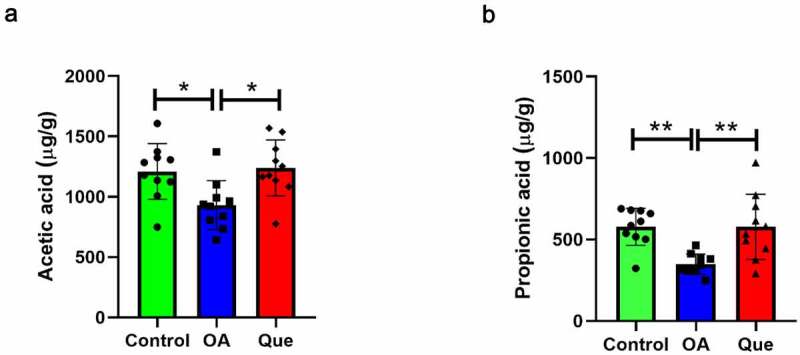
Quercetin increased SCFA levels. The levels of acetic acid (a) and propionic acid (b) were measured using GC-MS in the three groups. The values are presented as the means ± SDs, **P < 0.01 and *P < 0.05

One of the mechanisms responsible for the effects of the microbiota on the health of humans is SCFA production via the fermentation of dietary fiber. As a consequence, changes in the levels of SCFAs, including acetic, butyric, caproic, isobutyric, isovaleric, propionic, and valeric acids, in rat feces were studied and analyzed by GC-MS and other measurements. The primary SCFAs in rat feces were acetic, butyric, and propionic acid. Acetic acid (P = 0.0247, Figure 2a) and propionic acid (P = 0.0025, Figure 2b) were significantly lower in the OA group than in the control group, and acetic acid (P = 0.0122, Figure 2a) and propionic acid (P = 0.0025, Figure 2b) were significantly higher in the quercetin group than in the OA group, suggesting that quercetin promotes the production of SCFAs.

**Figure 3.**
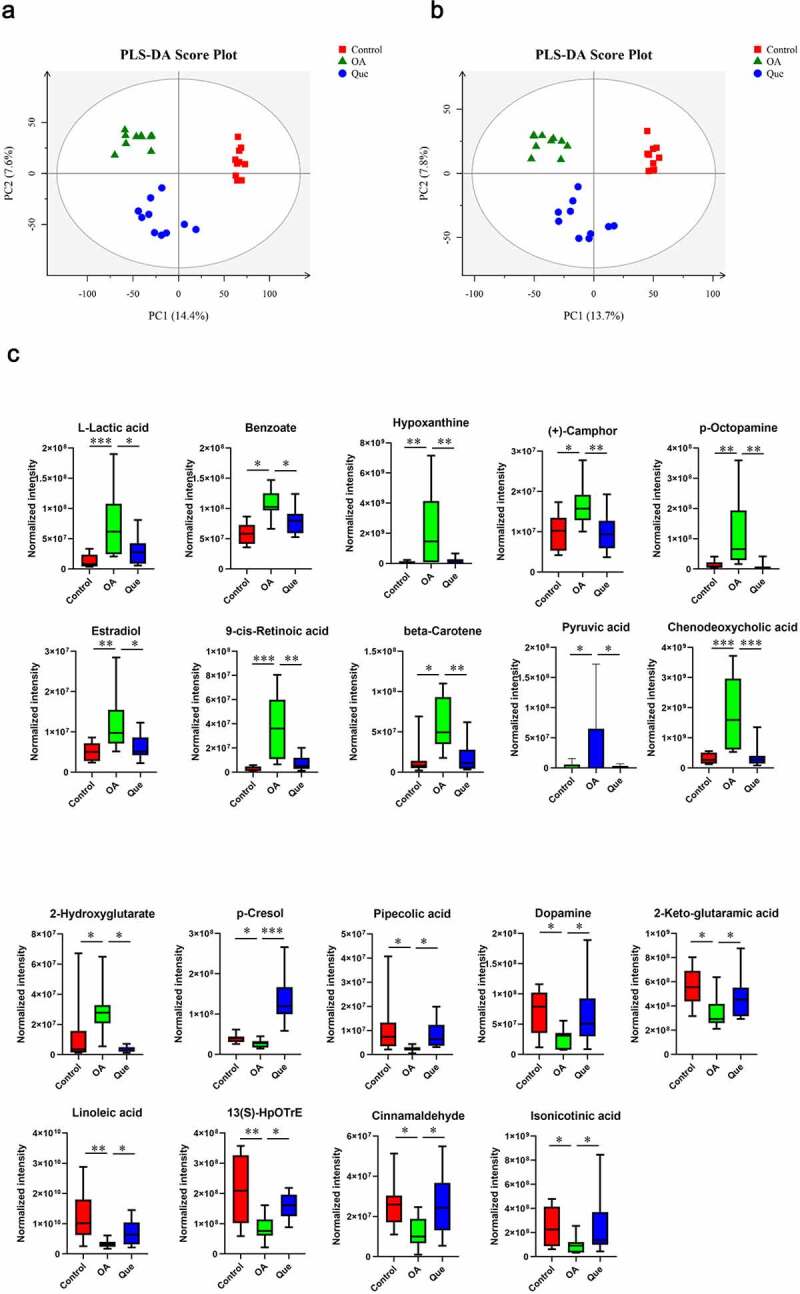
**Effect of disruption on the fecal metabolome**. Partial least square-discriminant analysis (PLS-DA) of MS data. (a) PLS-DA for positive ion mode data. (b) PLS-DA for negative ion mode data. (c) Fecal metabolite disorder in the three groups. The data are presented as the means ± SDs. Significant differences between groups are indicated by ***P < 0.001, **P < 0.01 and *P < 0.05

**Figure 3.**
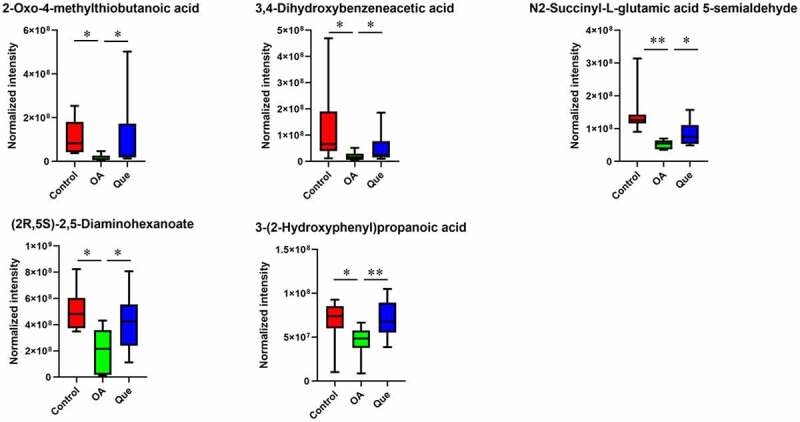
Continued

### Quercetin reversed perturbations in the metabolome of OA rats

The metabolomic profiles of the fecal contents of control rats, OA rats and quercetin-treated rats were compared using partial least square-discriminant analysis (PLS-DA) (Figure 3b) to visually separate the different groups. Many perturbed metabolites with diverse structures and biological functions were identified. We detected significant differences in metabolites among the three groups using MS/MS analysis. Our study found significantly dysregulated metabolites in the OA rats compared with the control rats. A total of 11 metabolites with increased levels and 13 metabolites with decreased levels were identified in the OA rats. However, these changes were significantly abrogated in quercetin-treated OA rats (Figure 3c), strongly suggesting changes in the features of the gut microbiota in the quercetin-treated OA rats.

### Association between the gut microbiota composition and the host metabolome

**Figure 4.**
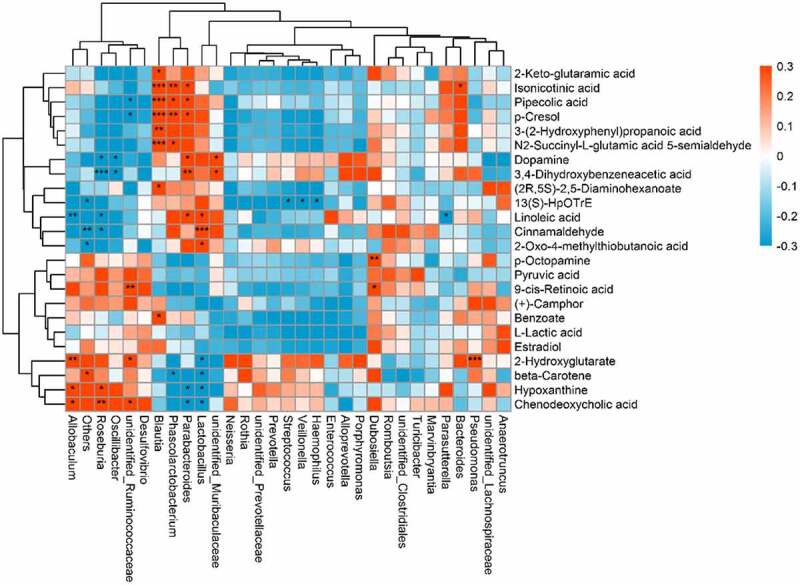
Relationship between the gut microbiome and host metabolome. Heat maps indicate positive (red) and negative (blue) correlations between the levels of host metabolites and the gut microbiome genera in quercetin-treated rats compared to OA rats. The legend shows correlation values from −1 to 1 and the associated colors: red for positive correlations, and blue for negative correlations (* P < 0.05, ** P < 0.01, *** P < 0.001)

An indirect potential influence of the gut microbiota composition on the host metabolome has been recently reported; however, the relevant mechanism remains unclear. Therefore, this correlation was further analyzed after quercetin treatment in OA rats (Figure 4). *Lactobacillus* was positively correlated with changes in 2-oxo-4-methylthiobutanoic acid, linoleic acid and cinnamaldehyde but was negatively correlated with changes in hypoxanthine, chenodeoxycholic acid, beta-carotene and 2-hydroxyglutarate. *Unidentified Ruminococcaceae* were positively correlated with changes in 9-cis-retinoic acid, chenodeoxycholic acid and 2-hydroxyglutarate and negatively correlated with changes in pipecolic acid. *Roseburia* was positively correlated with changes in chenodeoxycholic acid and hypoxanthine but negatively correlated with changes in 3,4-dihydroxybenzeneacetic acid, dopamine, linoleic acid and cinnamaldehyde. *Blautia* was positively correlated with changes in isonicotinic acid and pipecolic acid. *Phascolarctobacterium* was positively correlated with changes in isonicotinic acid and pipecolic acid but negatively correlated with changes in beta-carotene. *Parabacteroides* was positively correlated with changes in isonicotinic acid, pipecolic acid, 3,4-dihydroxybenzeneacetic acid and linoleic acid but negatively correlated with changes in chenodeoxycholic acid and hypoxanthine, which suggested that changes in gut microbiota features were correlated with the host metabolome changes under quercetin treatment.

## Discussion

As the most prevalent arthritis- and age-related degenerative disease, OA affects the joints with a complicated pathology involving inflammation and extracellular matrix (ECM) degradation. For most of the currently available therapeutic options, significant limitations remain, which raises a major concern for identifying novel therapeutic options **[**34**]**. Quercetin exerts anti-inflammatory activity in multiple different disease models **[**35–37**]**. Hu et al. **[**4**]** found that quercetin alleviated OA by attenuating the inflammation and apoptosis of chondrocytes. Feng et al. **[**38**]** found that quercetin attenuated oxidative stress-induced apoptosis via SIRT1/AMPK-mediated inhibition of ER stress in rat chondrocytes and prevented the progression of OA in a rat model. Quercetin is a member of the flavonoid family and has anti-inflammatory properties in degenerative diseases. Studies have suggested that quercetin is a promising treatment for OA. However, the effect of quercetin on the pathological process of OA is not very clear.

This study investigated the effects of quercetin on OA and delineated a potential mechanism. A combination of gut microbiota analysis, SCFA measurements and metabolomics profiling analysis was used to further assess the use of quercetin.

To our knowledge, joint disease caused by the gut microbiota has been previously reported in a few studies, and this causation could be classified into three types of interactions [39]. First, the gut microbiota partially regulates nutritional absorption in the gut. Second, the response from immune cells to microbial species in the gut further induces subsequent inflammatory effects. Third, a growing body of evidence linking the microbiome with OA pathogenesis is becoming available.

In this study, quercetin treatment induced dramatic changes in the gut microbiota in OA rats, such as increasing the proportion of *Clostridia* and decreasing the proportion of *Bacilli* (at the class level), which indicated that quercetin modulated the total microbial population in the gut. Quercetin treatment has also been found elevate levels of the genus *Lactobacillus* and deplete the genera in *unidentified Ruminococcaceae. Lactobacillus* in the gut has been suggested to have a beneficial effect on the stress response, depressive disorder and inflammation [40–42]. It has been reported that *Lactobacillus* can alleviate OA-associated pain and delay the progression of the disease by inhibiting proinflammatory cytokine production and reducing cartilage damage [43]. Others research has confirmed that *Lactobacillus* consumption could serve as a novel therapeutic option in the clinical management of knee OA, improving treatment outcome likely through reducing serum hs-CRP levels [44]. Others reported that the oral administration of *Lactobacillus* ameliorates the progression of osteoarthritis by inhibiting joint pain and inflammation in a rat model of OA [45]. These findings suggest the therapeutic potential of *Lactobacillus* in OA. Our results provide direct evidence that *Lactobacillus* counts are increased in OA rats treated with quercetin compared with controls, which supports the protective effect of *Lactobacillus* in OA. We hypothesized that quercetin treatment altered the observed fecal microbial signatures of OA rats.

Our study found that fecal samples from OA rats had lower SCFA production levels than the controls, particularly for acetic acid and propionic acid. Nevertheless, in the quercetin-treated group, the concentrations of acetic acid and propionic acid in fecal samples were significantly increased compared to those in the OA group. As fermentative products from the gut microbiota, these SCFAs primarily consist of acetic, butyric, and propionic acids, indicating different phyla of SCFA producers [46].

This study found changes in the gut microbiota and disruption of host metabolites in OA rats. We performed PLS-DA and found significant separation between samples based on individual variability. In comparisons between OA rats and control rats, a total of 11 and 13 metabolites were identified as significantly increased and decreased, respectively. As shown in Figure 3, L-lactic acid, benzoate, hypoxanthine, (+)-camphor, p-octopamine, estradiol, 9-cis-retinoic acid, beta-carotene, pyruvic acid, chenodeoxycholic acid and 2-hydroxyglutarate were higher in the OA group than in the control group. In contrast, the levels of p-cresol, pipecolic acid, dopamine (DA), 2-keto-glutaramic acid, linoleic acid, 13(S)-HpOTrE, cinnamaldehyde, isonicotic acid, 2-oxo-4-methylthiobutanoic acid, 3,4-dihydroxybenzeneacetic acid, N2-succinyl-L-glutamic acid 5-semialdehyde, (2 R,5S)-2,5-diaminohexanoate and 3-(2-hydroxyphenyl)propanoic acid were significantly lower in OA rats. Changes in the levels of these metabolites were significantly abrogated in quercetin-treated OA rats.

Estradiol is the main estrogen in pre- and postmenopausal women. The estrogen levels in joint fluid are correlated with the estrogen levels in the blood, and the estradiol levels in joint fluid are similarly correlated with the estrogen levels in sera from women with OA. Changes in the levels of hormones, such as estradiol, increase the incidence of OA **[**47**]**. Some metabolites with important regulatory functions were altered in the fecal samples of OA rats, but quercetin reversed these changes.

According to our findings, following exposure to quercetin, the relevant gut microbiota may causally interact with the host metabolome, which eventually leads to correlations between the gut microbiota and host metabolome. This study suggested that quercetin altered the gut microbiota and metabolome in OA rats and vice versa. Such findings may further aid in the development of novel biomarkers. However, there is no research on this mechanism yet. Further investigations of the mechanism of action are still required. We observed significant differences among the three rat groups, but the rat population was limited, and future work would increase the number of samples. Furthermore, it is necessary to discover the role and mechanism of the relationship between quercetin and intestinal epithelial cell permeability. Although further studies are required, based on the results presented here, quercetin is expected to have therapeutic potential in OA.

## Conclusion

Our study demonstrated that quercetin treatment significantly influences the features and composition of the gut microbiota as well as metabolism in OA rats. These findings are the first to suggest possible mechanisms by which quercetin affects gut microbiota-host metabolic homeostasis in OA rats, which is an important finding in the understanding of OA and thus will further aid in the relevant therapy and treatment.

## Highlights

Quercetin modulated changes in gut microbiota features in OA rats.

Quercetin increased SCFA levels in OA rats.

Quercetin reversed perturbations in the metabolome of OA rats.

Alterations in the gut microbiota and the host metabolome by quercetin treatment were correlated in PAH rats.
